# Artificial infestation of white-tailed deer with ticks (Acari: Ixodidae) to study tick–host interactions

**DOI:** 10.1093/jisesa/iead029

**Published:** 2023-05-23

**Authors:** Alec S Baker, Kelly A Persinger, Pia U Olafson, Tammi L Johnson

**Affiliations:** Department of Rangeland, Wildlife and Fisheries Management, Texas A&M University, 495 Horticulture Road, College Station, TX 77843, USA; Texas A&M AgriLife Research and Extension Center, 1619 Garner Field Road, Uvalde, TX 78801, USA; Texas A&M AgriLife Research and Extension Center, 1619 Garner Field Road, Uvalde, TX 78801, USA; Livestock Arthropod Pests Research Unit, USDA-ARS, 2700 Fredericksburg Road, Kerrville, TX 78028, USA; Department of Rangeland, Wildlife and Fisheries Management, Texas A&M University, 495 Horticulture Road, College Station, TX 77843, USA; Texas A&M AgriLife Research and Extension Center, 1619 Garner Field Road, Uvalde, TX 78801, USA

**Keywords:** artificial infestation, captive, tick, tick–host, white-tailed deer

## Abstract

White-tailed deer (*Odocoileus virginianus*) are a main host for the adult life stages of tick species of medical and veterinary importance. Since white-tailed deer play a vital role in tick ecology, research has been conducted to understand this tick–host relationship. To date, research involving captive white-tailed deer and artificial infestation of these animals with ticks has focused on host suitability, the role of white-tailed deer in tick-borne diseases, and anti-tick vaccine research. The methodology reported for these studies was at times not descriptive and inconsistent regarding how and what region of the white-tailed deer was infested with ticks. Here, we propose a standardized method to artificially infest captive white-tailed deer with ticks for research purposes. The protocol describes a method proven effective to experimentally infest captive white-tailed deer with blacklegged ticks (*Ixodes scapularis*) to study tick–host interactions. The methods can be reliably transferred for experimental infestation of white-tailed deer by other multi-host and one-host tick species.

## Introduction

White-tailed deer (Odocoileus virginianus Zimmermann) (Artioda­ctyla: Cervidae) are the host of various arthropod ectoparasites, including ixodid and argasid ticks, *Demodex* spp. and *Psoroptes* spp. mites in the order Trombidiformes and Sarcoptiformes, respectively, deer keds (Diptera: Hippoboscidae), and sucking and chewing lice in the order Phthiraptera (Kellogg et al. 1971, Poh et al. 2022). In particular, blacklegged (Ixodes scapularis Say) (Acari: Ixodidae), lone star (*Amblyomma americanum* Linnaeus) (Acari: Ixodidae), and other tick species of medical and veterinary importance (see Table 1) use white-tailed deer (WTD) as a main host for the adult life stages (Rand et al. 2000, Stafford III and Williams 2017, Tsao et al. 2021). As such, WTD play a vital role in the reproductive success of these tick species. While tick larvae and nymphs also feed on WTD, very little is known about the importance of this host as a bloodmeal source for immature life stages (Stafford III and Williams 2017).

**Table 1.** Tick species of medical and veterinary importance that have been documented infesting white-tailed deer in North America

Tick species	Reference
*Amblyomma americanum*	Bolte et al. 1970, Kellogg et al. 1971
*Amblyomma cajennense*	Samuel and Trainer 1970
*Amblyomma inornatum*	Cook et al. 1969, Samuel and Trainer 1970
*Amblyomma maculatum*	Samuel and Trainer 1970, Kellogg et al. 1971
*Dermacentor albipictus*	Kellogg et al. 1971, Machtinger et al 2021
*Dermacentor andersoni*	Schofield and Saunders 1987
*Dermacentor nigrolineatus*	Kellogg et al. 1971
*Dermacentor nitens*	Kellogg et al. 1971
*Dermacentor variabilis*	Kellogg et al. 1971
*Haemaphysalis leporispalustris*	Kellogg et al. 1971
*Haemaphysalis longicornis*	White et al. 2021
*Haemaphysalis juxtakochi*	Keirans and Restifo 1993
*Ixodes affinis*	Harrison et al. 2010
*Ixodes scapularis*	Kellogg et al. 1971
*Otobius megnini*	Russell 1967
*Rhipicephalus annulatus*	Pound et al. 2010
*Rhipicephalus microplus*	Kellogg et al. 1971
*Rhipicephalus sanguineus*	Kellogg et al. 1971

White-tailed deer influence the abundance and geographic spread of tick populations, but they alone cannot support the enzootic cycles for many of the known tick-borne pathogens of medical and veterinary importance (Tsao et al. 2021). For example, WTD are not a competent reservoir host for *Borrelia burgdorferi* sensu stricto, the pathogen causing Lyme disease, and they may even clear the pathogen from attached ticks (Telford et al. 1988, Roome et al. 2017). Oliver et al. (1992) described the inability of *I. scapularis* to acquire *B. burgdorferi* from WTD inoculated with the bacterium, further determining the role of WTD in the Lyme disease system. However, WTD are the main reservoir host of *Ehrlichia chaffeensis* and *E. ewingii*, the pathogens that cause human monocytic ehrlichiosis (Paddock and Yabsley 2007). Habitats favored by WTD also support populations of other wildlife species which can be hosts for immature life stages of ticks and may be reservoirs for various disease pathogens (Tsao et al. 2021).

Since WTD play a vital role in tick ecology, numerous studies have focused on experimental infestation in a captive setting (see Table 2). Davey (1990) described suitability of WTD as hosts for *Rhipicephalus* (*Boophilus*) *annulatus* (Acari: Ixodidae). Koch (1988) described the suitability of WTD as hosts for *A. americanum*. Multiple studies infested captive WTD with ticks to determine their role in transmitting and serving as a reservoir host for *E. chaffeensis* and *E. ewingii* (Ewing et al. 1995, Kocan et al. 2000, Varela-Stokes 2007a, 2007b). Kuttler et al. (1971) examined the inability of *R. (B.) annulatus* to transmit anaplasmosis to WTD, while Barker et al. (1973) studied the effect of *Theleria* spp. on WTD fawns infested with infected *A. americanum*. Furthermore, a single study delivered an anti-tick vaccine to WTD for population reduction of *R.* (*B.*) *microplus* (Carreón et al. 2012). While these studies all involved artificially infesting captive WTD with ticks, the methodology reported within these studies at times lacked sufficient detail for replication and varied in how and what region of the WTD was infested with ticks.

**Table 2.** Published literature of white-tailed deer (WTD) experimentally infested with different tick species and life stages for research purposes

Reference	Tick species	Tick life stage(s) used^a^	Region of WTD infested
Oliver et al. 1992	*Ixodes scapularis*	L, N	Side of animal
Ewing et al. 1995	*Amblyomma americanum*	L, N, A	Not restricted
Kuttler et al. 1971	*Rhipicephalus annulatus*	L	Not specified
Reichard et al. 2009	*Ixodes scapularis*	N	Not specified
Varela-Stokes 2007b	*Amblyomma americanum*	A	Back of animal
Barker et al. 1973	*Amblyomma americanum*	A	Head/neck
Yabsley et al. 2008	*Amblyomma americanum*	N	Back of animal
Varela-Stokes 2007a	*Amblyomma americanum*	N, A	Back of animal
Davey 1990	*Rhipicephalus annulatus*	L, N, A	Not restricted
Kocan et al. 2000	*Amblyomma maculatum*	N	Not Restricted
Carreón et al. 2012	*Rhipicephalus microplus*	L, N, A	Back of animal
Koch 1988	*Amblyomma americanum*	L, N A	Not Restricted

^a^L, larvae; N, nymphs; A, adults.

Previous artificial infestation studies reported in the literature had various methods and location for infestation, and the decision to either restrict of allow host grooming was each dictated by individual study objectives and Table 2. Some studies free-release ticks to infest WTD (Davey 1990, Kocan et al. 2000), while others restricted the infestation to the back of the animal in a tick tube/chamber (Varela-Stokes 2007a, 2007b, Yabsley et al. 2008, Carreón et al. 2012). One study restricted the infestation to the head and neck regions of the WTD (Barker et al. 1973), and others did not specify if the tick infestation was restricted to a certain body region (Kuttler et al. 1971, Ewing et al. 1995, Reichard et al. 2009).

A better understanding of the interaction between WTD and ticks is needed to inform and evaluate methods targeting this host for tick control and to better understand the role of WTD in tick-borne disease systems. There is currently no standardized method to experimentally infest WTD with ticks and other ectoparasites of interest with the intention of preventing grooming. This provided the opportunity to propose a method for artificially infesting captive WTD with ticks for research purposes. The protocol below describes a method proven effective to experimentally infest captive bottle-raised WTD with *I. scapularis* to study tick–host interactions.

## Experimental Design

Female WTD fawns ranging in age from 2 to 6 days old were bottle raised for 16 weeks at the [blinded for review]. Bottle raising allowed the animals to become accustomed to interacting with people and helped reduce their stress while being handled to develop this procedure. A lift chute designed to accommodate juvenile and adult WTD was used to securely raise the animal off the ground to restrict movement during manipulations. For at least three weeks prior to infestation, deer were acclimatized to being herded into an alley, which led to a small building with a room leading to the chute. This area was left open for the deer to explore for several days, following which deer were herded through the entire handling process without any actual manipulations, to include allowing them to enter and exit through the chute at ground level. Habituating the deer to this movement process was beneficial in establishing a routine pattern. All animal usage was approved by the [blinded for review] Animal Care and Use Committee protocols #2020-018A (approved 06/24/2020) and #2020-028A (approved 10/20/2020) and animal care was in accordance with our Program of Veterinary Care.

We experimented with different cotton stockinette, hereafter tick patch, widths, and lengths that would contain a minimum of 100 adult *I. scapularis* and be long enough to tie a knot for patch closure. We selected the area at the base of the neck between the shoulders for placement of the tick patch to reduce the ability of WTD to disturb the patch area. The area between the front shoulders was shaved down to the skin before applying adhesive and tick patch to the WTD. We experimented with placing plastic Elizabethan collars (E-collars; Dogswell Remedy & Recover rigid E-collar, Chesterfield, MO) around the animal’s neck to prevent them from disturbing the tick patch. Because the size of the E-collar used varied depending on the size of the WTD, we had medium, large, and extra-large size E-collars on hand. We applied a strip of Velcro self-adhesive Hook and Loop fastener to a section of the E-collar to help reinforce the E-collar’s plastic snap closures and to minimize the ability of the WTD to easily remove the E-collar.

Once the tick patch was applied to the WTD, the animals were housed in individual pens during the experimental infestation to prevent inter-host grooming. The individual pens (132 m^2^) with 2.4 m walls were located in an open-sided, covered barn that provided exposure to natural light. Pen design was in accordance with the “Guide for Care and Use of Agricultural Animals in Research and Teaching 4th Edition” (2020). Pens were cleaned every 2–3 days, and WTD were observed twice a day for temperament and body condition and to ensure ample access to food and water. These observations were also done to ensure that the E-collar and “tick patch” were intact and not disturbed. In the event the WTD was able to remove the E-collar and/or untie the “tick patch” the animal was restrained, the E-collar was refitted, and/or the “tick patch” was tied closed.

We tested INSTAbond AD-80 (ACCRAbond, Inc), Kamar adhesive glue, and liquid bonding cement (TORBOT Group, Inc) to identify which adhesive would work best to secure the tick patches to the WTD for at least the length of time needed for *I. scapularis* to feed to repletion. *Ixodes scapularis* adult females take 6 to 11 days to feed to repletion (Troughton and Levin 2007, Kocan et al. 2015). To test each adhesive, we applied tick patches to 2 WTD with each of the 3 adhesives (*N* = 6 WTD total), and we monitored the patches for 15 days. Each product was evaluated for its longevity of adhesive quality, ease of application/removal, and degree of irritation to the animal.

Once an ideal tick patch size and adhesive were identified, we further developed the procedure to infest WTD with adult male and female *I. scapularis*. Eight female WTD were infested with 100 *I. scapularis*; for each WTD, 50 males and 50 females were pre-mated in 50-ml conical centrifuge tubes for 24 h prior to infestation (Oklahoma State University Tick Rearing Laboratory). The integrity of the tick patch was checked daily after application to ensure no breaches or disturbances, for example, evidence of chewing, licking, or rubbing. Beginning 5 days after infestation, WTD were run through the chute, the patch was untied and checked for detached fed female ticks. At this time, any needed repairs to the tick patch were also made. This daily monitoring allowed us to determine the number of days to the start of rapid feeding for female ticks and the potential duration of time for all ticks to feed to repletion as part of an experimental tick infestation on WTD.

## Materials

The materials needed for this procedure are summarized in Table 3. We recommend the use of a blindfold for animals to help reduce their stress when being handled. For tick patch application, the following items are needed: a ~100-mm-wide cotton stockinette cut into ~300-mm-long sections (one per WTD), hair clippers (for livestock) to shave the area prior to applying the tick patch, ~25 mm wide paint brushes, and the adhesive. The quantity of ticks, species, and life stage(s) of interest will vary depending on individual research objectives. Regardless of tick species and life stage being used, ticks should be separated into individual vials (one vial per WTD). The size of the vial will depend on quantity of ticks being used, for example, for 100 adult *I. scapularis*, we used a 50 ml conical centrifuge tube with 10 air holes created in the screw-capped lid (punctured the lid using a 18G hypodermic needle) to house ticks prior to infestation. Fine tip forceps can be used to transfer ticks from the vial to the tick patch when needed. One E-collar per animal (size of WTD dictates the size of E-collar) is needed to minimize the impact of licking and chewing on the tick patch. Feather forceps are ideal for transferring replete adult female ticks from the tick patch to a plastic vial (one vial per WTD). Given the size of replete female ticks, we used a 50-ml conical centrifuge tube with 10 air holes created in the lid to collect engorged, female ticks. We recommend using fine tip forceps if collecting partially fed or semi-engorged ticks from the host, as it allows for better grip of the tick’s mouthpart. Depending on the life stage, species, and quantity of ticks being collected from the WTD, the size, number, and type of vials used for collection will vary. To remove the tick patch, at the end of an infestation, a squirt bottle of adhesive remover will be needed. Not included in Table 2 are a chute (lift or drop) designed to handle WTD and a facility with individual pens to house the animals during tick infestations. The chute used for optimizing this protocol was constructed by the US Department of Agriculture, Agricultural Research Service (Kerrville, TX) (Pound 2003), and modified to accommodate deer of various sizes from 6 to 7-month-old fawns to adults. Similar adjustable chutes are commercially available and can be acquired from vendors. The number of individual pens will vary depending on research objectives.

**Table 3.** Materials needed to artificially infest white-tailed deer with ticks

Item	Item #	Cost	Quantity needed
Half Face Hood X-Small	HD01-XS	$15.95	1
Half Face Hood Small	HD01-S	$17.95	1
Tubular Stockinette Bandage	B075M1B2PG	$29.99	1 Roll
Clippers	13084-1	$208.95	1
#30 Clipper blade	1204442	$39.99	2
Chip Paint Brushes (25 mm)	B078XK3KTV	$26.99	1 Package (96)
TORBOT Bonding Cement	TR410	$41.89	1 Package (5)
Fine Tip Forceps	UX-07387-08	$13.30	2 Pair
Featherweight Forceps	B07B4767WR	$13.99	2 Packages (4)
50 ml Centrifuge tubes	21008-169	$388.27	1 Case (500)
Elizabethan Collars	2433592	$17.99	1/animal
TORBOT Solvent Adhesive Remover	TR420	$18.79	2
Squirt Bottle	6FAV1	$8.07	2

## Protocol

The protocol described here is a standardized method to be used on captive WTD in research environments to study tick–host interactions. The protocol was optimized using tame to semi-tame, bottle raised WTD, and was designed to limit the impact of host grooming throughout an artificial tick infestation. We have used this protocol for both juvenile and adult WTD from a herd that we established (bottle raised), and this herd was continuously habituated to human interaction and acclimated to the chute prior to research activities commenced. Relevant protocols for bottle rearing WTD fawns are available (see Kendall et al. 1998, Palmer et al. 2017, Stutts et al. 2018).

### Patch Attachment

**Fig. 1.**
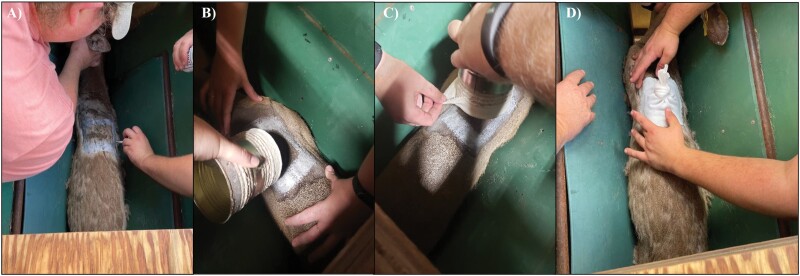
Tick patch application to back of white-tailed deer. A) Applying adhesive to white-tailed deer. B) Preparing to apply tick patch to white-tailed deer. C) Applying tick patch to white-tailed deer. D) Tick patch on white-tailed deer.

We recommend patch application be completed one day prior to infesting WTD with ticks to observe animal reaction. This allows time for the adhesive to set and for additional adhesive to be applied if needed. A single WTD is processed at a time. Once the animal is in the chute, it is blindfolded, and hair clippers are used to shave an approximately 152 mm^2^ square area at the base of the neck between the shoulder blades of the deer. Once the patch area is completely shaved, all shaved hair is brushed away to prevent interference when applying the adhesive and stockinette. Using a ~25-mm paint brush, 3 layers of liquid bonding cement are applied to the shaved area in a square outline (approximately 102–127 mm^2^), and to the bottom edge (13–25 mm wide) of a pre-cut ~305-mm-long cotton stockinette following product instructions. Each layer should dry until slightly tacky when touched before applying the next layer. The cotton stockinette patch is then applied to the animal once the liquid bonding cement (TORBOT Group, Inc) on both the WTD and stockinette is tacky (Fig. 1). After the tick patch is confirmed to be adhered to the WTD, an E-collar is fitted on the WTD before releasing the animal from the chute, and transporting the animal to its individual pen. Animals are transported individually to their pens in a 1.2 m × 1 m × 1.4 m game translocation crate mounted on a metal frame with wheels.

### Experimental Tick Infestations

**Fig. 2.**
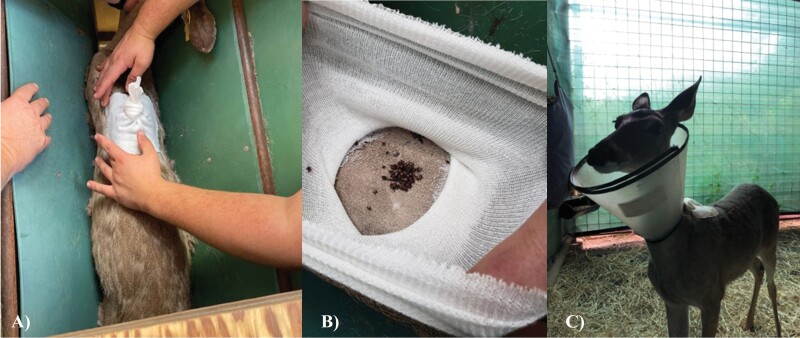
Infesting white-tailed deer with adult blacklegged ticks. A) Inspecting tick patch to ensure no breaches are present before applying ticks. B) Adult blacklegged ticks dispensed into tick patch. C) White-tailed deer in individual pen wearing Elizabethan collar after being infested with ticks.

One day after adhering the tick patch, each WTD is run into the chute and blindfolded. The tick patch is examined to ensure there are no breaches in the patch prior to infesting with ticks. If small breaches in the tick patch are found, additional liquid bonding cement should be applied before infesting. If no breaches in the tick patch are found, the tick vial is inverted and tapped to dispense ticks within the tick patch, after which the tick patch is tied to close. Once the patch is tied closed, an E-collar is placed around the animal’s neck, and the WTD is released from the chute and transported to its individual pen (Fig. 2). The life stage and species of tick used will impact how frequently the WTD will need to be checked for replete ticks. We recommend checking the tick patch daily to ensure maintenance of patch integrity and if unsure when rapid engorgement occurs. When checking for replete ticks, the WTD should be handled in the chute, as described above. One 50-ml conical per deer and feather forceps should be used to collect replete ticks from tick patch.

### Patch Removal

Once all ticks feed to repletion and/or are removed from the WTD, TORBOT Solvent Adhesive Remover is applied (via a squeeze bottle) to the edges of the patch. After 30–60 s the edge of the patch should easily peel off the WTD. If the tick patch does not easily peel away in 60 s, apply more adhesive remover. If skin irritation has occurred at the site of patch attachment or tick bite site, topical antibiotic ointment can be applied. All used tick patches should be discarded appropriately, making sure that all surviving male ticks are also collected and disposed. E-collars should be sanitized and can be reused. Discard of any biohazardous material via autoclaving or incineration, as outlined in approved safety protocols obtained from institutional regulatory committees.

## Results

**Fig. 3.**
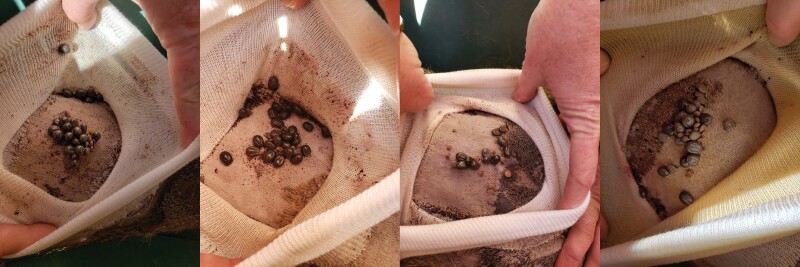
Adult blacklegged ticks within tick patches of four different white-tailed deer at 5 days after infestation.

Tick patches applied using INSTAbond AD-80, Kamar adhesive glue, and liquid bonding cement remained adhered for the duration of the 15-day patch monitoring period. INSTAbond AD-80 and liquid bonding cement were both easy to apply with paint brushes, but the tacky consistency of Kamar adhesive glue made it difficult to apply. The liquid bonding cement and Kamar adhesive glue did not cause any skin irritation upon application or during the monitoring period, while skin irritation during application and during patch removal was evident using INSTAbond AD-80. Due to the ease of application and lack of skin irritation to the animal, we elected to use liquid bonding cement for our tick infestation protocol. After experimentally infesting 8 WTD with ticks, we were able to successfully collect replete female ticks from the tick patch over a 5- to 11-day period post-infestation (Fig. 3). The percentage of female ticks that fed to repletion per WTD ranged from 58 to 98%.

## Discussion

Here we present a standardized protocol to artificially infest juvenile and adult WTD with ticks, allowing for the direct comparison between results of future research projects. The WTD we used to develop this protocol were 6–7 months old, and these same animals were again infested using this protocol when they were over a year old and full adult size. A critical component of this protocol is the investment in developing a herd of WTD that are bottle raised and continuously habituated to human interaction. Potential limitations of this protocol include the longevity of adhesion, especially if using this method to infest with one-host tick species that have upwards of a 30-day on-host period. Since we only tested the adhesive over a 15-day period, a trial run without ticks on the animal should be conducted to verify whether liquid bonding cement will last for longer periods of infestation or if another adhesive will need to be evaluated.

The weave of the stockinette should also be considered if infesting WTD with immature life stages of ticks. To ensure that immature ticks cannot escape the patch area, a finer weave fabric (e.g., muslin cloth; Cooksey et al. 1989) could be used. Surveys of hunter-harvested WTD indicate that ticks were primarily found on the head and front portion of these hosts (Poh et al. 2022). This could potentially be due to the foraging behavior of WTD, the reduced ability to groom the chest and inner ears with great efficiency, or the large amount of highly vascularized tissue on the ears that make these sites particularly ideal for tick attachment. We selected the base of the neck on the back of the WTD to limit the host’s ability to disturb the area of the tick infestation and ease of accessing the area when the animal is in the chute, but it is unclear if tick feeding efficiency differs depending on the body region. A comparison of tick feeding using our method and a method restricting tick feeding to the head region (similar to Barker et al. 1973) may clarify this, although we suspect that preferred feeding sites for ectoparasites may be a result of avoiding host grooming behaviors.

This protocol provides a mechanism to study the effectiveness of deer-targeted tick intervention strategies in a controlled, captive environment, as researchers can directly observe tick–host interactions. A better understanding of this interaction is critical for further defining the importance of WTD in the role of tick-borne disease systems, and this protocol can be used for studies involving experimental infection of the host with pathogens to monitor tick acquisition and/or transmission and host reservoir status. Palmer et al. (2017) define the requirements and recommendations for using WTD in such studies that require facilities with varying levels of biological safety certification. This protocol can also be adapted to investigate the interactions between WTD and other ticks of importance including *A. americanum* and the winter tick (*Dermacentor albipictus*) as well as additional vectors of medical and veterinary importance that utilize this host, including biting midges and deer keds.
